# The MediVoice implementation journey: ambient artificial intelligence for clinical documentation

**DOI:** 10.3389/fdgth.2026.1764465

**Published:** 2026-04-17

**Authors:** Jennifer Sumner, Yee Wei Lim, Timotius Tan, Andrew Makmur, Peter Forbes, Jason Phua, Lit Sin Quek, Clara Lee Ying Ngoh

**Affiliations:** 1 Centre for Research in Health System Performance (CRiHSP), Yong Loo Lin School of Medicine, National University of Singapore, Singapore; 2 Alexandra Research Centre for Healthcare in a Virtual Environment (ARCHIVE), Department of Healthcare Redesign, Alexandra Hospital, National University Health System, Singapore; 3 Department of Medicine, Yong Loo Lin School of Medicine, National University of Singapore, Singapore; 4 Transformation Office, National University Health System, Singapore; 5 AIO Innovation Office, National University Health System, Singapore; 6 Group Digital Office, National University Health System, Singapore; 7 Division of Respiratory, Sleep, and Critical Care Medicine, Department of Medicine, Alexandra Hospital, National University Health System, Singapore; 8 Division of Respiratory and Critical Care Medicine, Department of Medicine, National University Hospital, National University Health System, Singapore; 9 Emergency Medicine, Ng Teng Fong General Hospital, Singapore; 10 Group Technology Office, National University Health System, Singapore; 11 Department of Medicine, National University Hospital, Singapore; 12 Division of Biomedical Informatics, Yong Loo Lin School of Medicine, National University of Singapore, Singapore

**Keywords:** ambient, artifical intelligence (AI), healthcare (MeSH), implementation, scribe

## Abstract

Healthcare systems are increasingly turning to ambient Artificial Intelligence (AI) scribes to reduce documentation burden and lighten clinicians' cognitive load. In this brief research report, we introduce MediVoice, an ambient AI scribe developed and implemented within the National University Health System, Singapore. MediVoice was piloted across multiple clinical settings and rapidly evaluated through Plan–Do–Study–Act cycles. Doctors, nurses, and allied health professionals assessed its usability, accuracy, workflow fit, and potential time savings. Real-time feedback informed iterative refinement, enabling organisational learning and reinforcing the value of experimentation during early AI adoption. Broader deployment required leadership engagement, an AI community of practice, role-specific training, user champions, recognition of its value, and supporting digital and organisational infrastructure. Looking ahead, routine use will require integration with the electronic medical record, enhanced speech recognition capabilities, and robust AI governance frameworks. MediVoice's trajectory shows that while ambient AI scribes can offer meaningful benefits, success requires more than technical capability. Effective implementation needs continuous adaptation, workflow alignment, cross-professional engagement, governance, and organisational readiness. This case study offers practical lessons for health systems seeking to introduce ambient AI tools within clinical environments.

## 1 Introduction

The sustainability of healthcare is a major challenge as clinical demand rises with ageing populations, and staffing shortages hamper service delivery (1, 2). Compounding these issues are the substantial documentation burdens placed on clinicians, which further exacerbate staffing-level issues by contributing to burnout (3). Addressing administrative load has consequently been a key priority for health systems, with Artificial Intelligence (AI) scribes being the most recent potential solution (4). AI scribes work by combining speech recognition technology to convert speech to text with large language models (LLMs) to generate structured summaries (5–8). Early evaluations of AI scribes suggest these systems may reduce perceived cognitive load and improve the work experience. However, actual reductions in documentation time and improvements in documentation quality are less clear (5–10). While these studies highlight the potential of AI scribes, there is still much to learn about the practicalities of developing and implementing these systems into clinical practice.

## 2 Context and programme description

**Figure 1:**
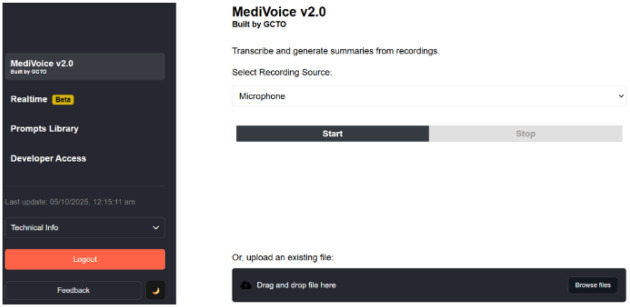
Screenshot of MediVoice interface.

In early 2024, the National University Health System (NUHS) embarked on efforts to identify and deploy an ambient AI system. Employing over 18,000 staff, NUHS is one of Singapore's three public healthcare clusters, serving the western region through three general hospitals, three specialty centres, two emergency departments, one urgent care centre, one community hospital, and seven polyclinics (11). Due to Singapore's strict regulatory framework, which requires storage of identifiable health data within accredited local infrastructure, commercial AI vendors were ruled out (12). Instead, an in-house application ‘MediVoice' was developed and deployed in September 2024. MediVoice is a multi-lingual ambient AI scribe (Figure 1) leveraging speech recognition technology from the Amazon Web Services Transcribe tool and the LLM Claude 3.5. The system is hosted locally within the Singapore Healthcare Commercial Cloud, an internet separated AWS virtual private cloud located in Singapore. Upon log-in, users can decide whether to generate a verbatim transcript or summaries mapped to standardised templates.

Pilot testing of MediVoice commenced in October 2024. MediVoice was initially deployed as a web-based platform. Users are required to log-in to activate the transcription function. Following that, users review the transcript, edit as needed, and transfer the text to the medical record, where documentation is signed off. The web-based approach helped to enable rapid deployment and evaluation using an agile quality improvement approach, specifically, the Plan–Do–Study–Act (PDSA) cycle framework (Table 1) (13). The PDSA approach facilitated rapid testing, continuous learning, and adaptive development within real-world clinical contexts. Through testing, real-time feedback helped to iterate and improve the product. Simple changes were made after evaluation cycles, while more complex changes were accumulated and deployed in a new version (i.e., MediVoice 2.0).

**Table 1:** PDSA cycles for mediVoice pilot.

Cycle	Use case tested	Test duration	Testing participants	Data collected
1	Family update (inpatient)	1 week	6 physicians	User uptake and use, Retention User experience
2	Comprehensive Geriatric Assessment (inpatient)	2 weeks	8 physicians	User uptake and use, Retention, User experience, Accuracy, Time savings
3	Nursing scribing tasks (inpatient)	4 weeks	29 nurses	User uptake and use, Retention, User experience, Time savings
4	Mixed-use scenarios (inpatient and outpatient)	4 weeks	30 users	User uptake and use, Retention, User experience, Accuracy, Time savings

A multidisciplinary project team, comprising sponsors, a project lead, a project manager, a technical developer, and outreach and education leads, provided oversight throughout the pilot. During each PDSA cycle, participants were recruited and trained individually on how to use MediVoice in their daily practice. Reminders were sent to participants, and an automatic dashboard tracked user activity and key performance metrics. Each of the four testing cycles lasted between 1 and 4 weeks, during which time the project team met to review findings and determine refinements. The evaluation captured data on process outcomes (uptake, retention, and time savings), documentation outcomes (accuracy, quality, and user satisfaction with generated notes), and balancing measures (potential workflow disruptions, unintended burdens, and safety risks).

## 3 Results

### 3.1 Lessons from pilot work

**Figure 2:**
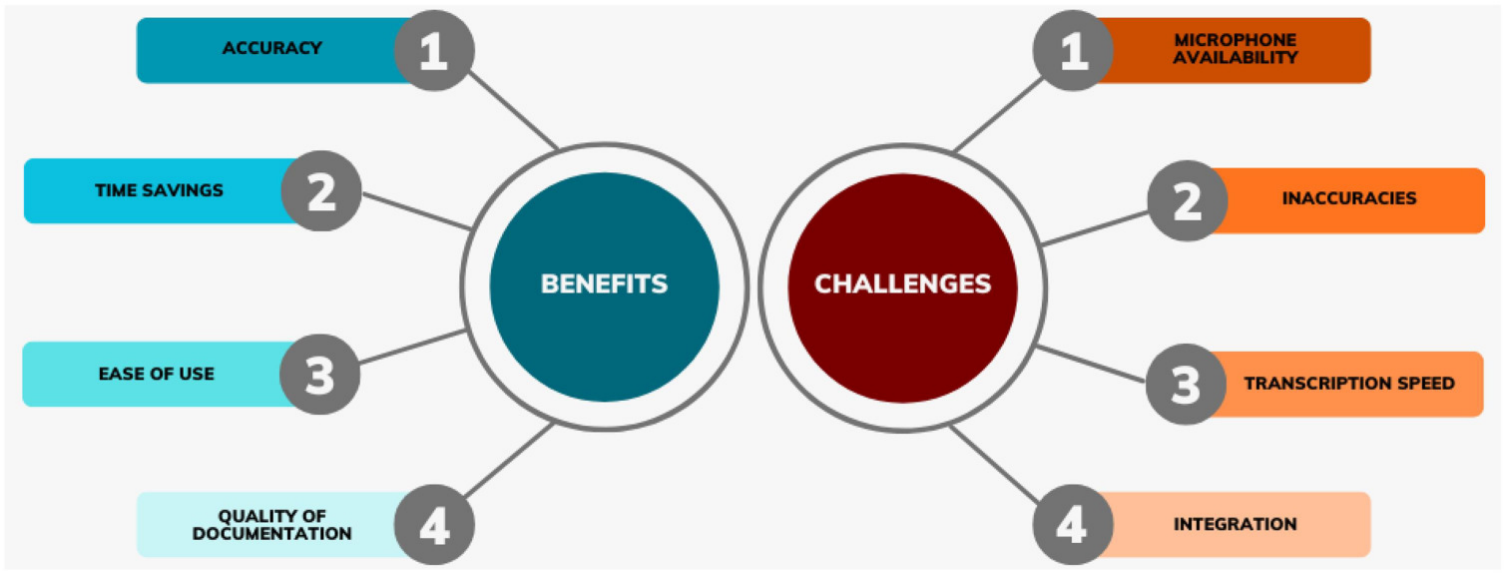
Summary of the barriers and enablers learnt from the MediVoice pilot.

The pilot surfaced several lessons (Figure 2). Users generally found MediVoice easy to use and that it generated accurate transcripts. However, while the system performed well in English, Mandarin and Malay, the quality declined in other local dialects such as Hokkien and Cantonese. Multilingual consultations and discussions with mask-wearing posed further challenges for the system. Early versions also suffered from slower transcription speeds, which improved in later iterations of MediVoice. Despite these issues, users felt the system saved them time, particularly during complex, unstructured conversations. Conversely, MediVoice was perceived as less useful in short consultations or in settings with existing Electronic Medical Record (EMR) templates or built-in phrasing functions, reducing the added value of an AI scribe.

Anecdotally, MediVoice also appeared to improve documentation quality by facilitating information recall and more accurately capturing data. In particular, staff whose native language was non-English shared that they used the tool as linguistic support to improve the clarity and completeness of their documentation. Sustained adoption, however, was viewed as contingent on seamless integration with the EMR and resolution of restricted microphone access in clinical environments.

Uptake was not uniform: we found nurses and allied health professionals adopted the tool more readily than physicians. Through informal user dialogues, this difference appeared to stem from how professionals engage with patients and document their encounters. For example, non-proceduralist doctors often multitask during consultations, typing notes directly into EMR templates. In contrast, a proceduralist, such as a surgeon, found greater value in the AI scribe, as their physical interaction with patients limits simultaneous documentation. Similarly, nurses, who often undertake physical assessments and document after encounters, viewed the MediVoice scribe as time-saving and helpful for information recall. Psychiatrists also benefited from the tool's ability to capture lengthy, nuanced conversations that do not easily fit standard templates.

### 3.2 Moving from pilot to mainstream deployment

**Figure 3:**
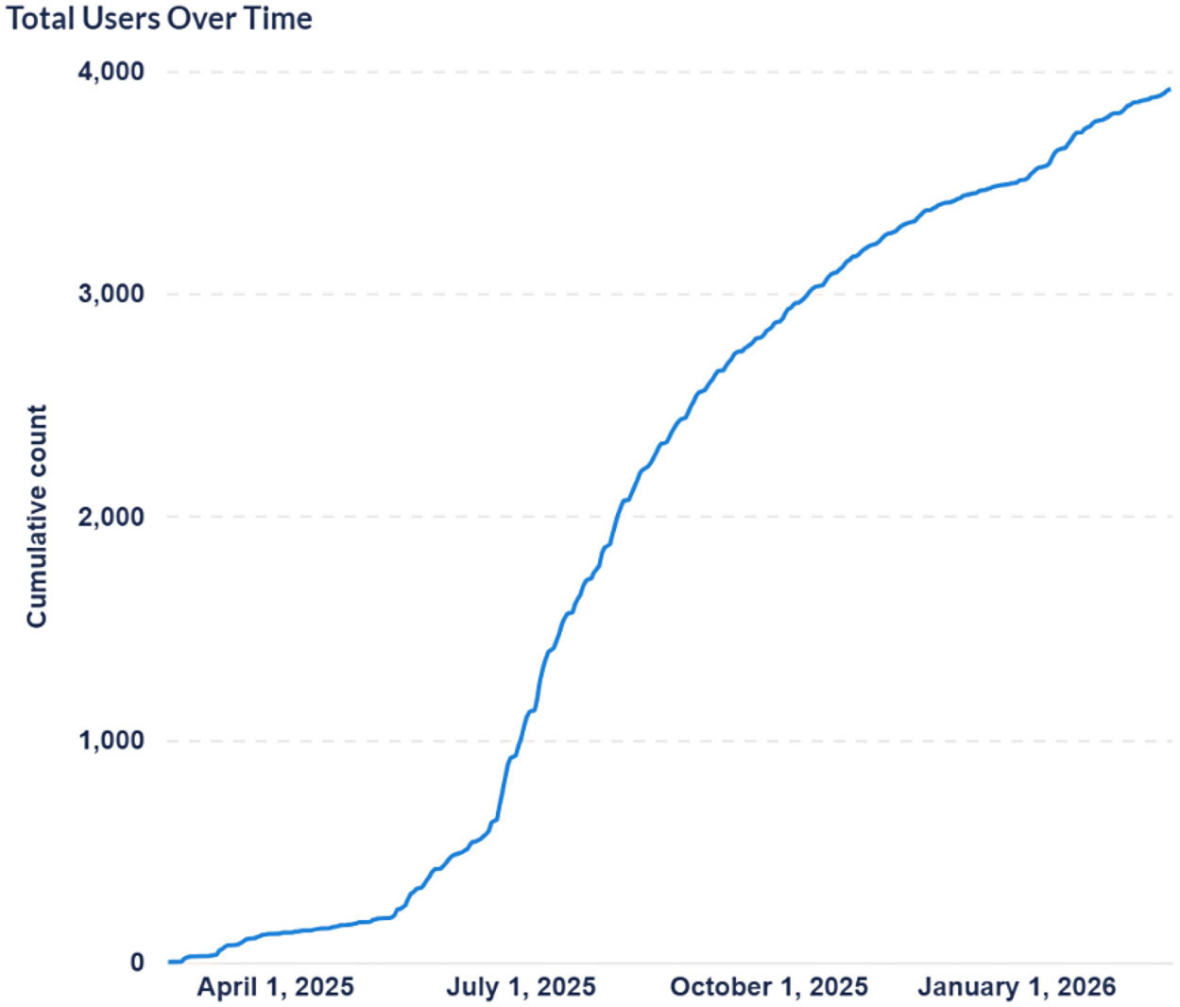
Total MediVoice users over time, from April 2025 to February 2026.

Following the pilot phase, adoption increased steadily, reaching 3,919 users by February 2026 (Figure 3). To understand who was using the system during scale-up, we examined user profiles at an earlier phase (August 2025). At that time, MediVoice had reached 1048 users. We found that uptake was dominated by specific professional groups: 43% administrative and ancillary staff (primarily using the system for meeting minutes), 30% nursing staff, 18% pharmacists or allied health professionals and 9% physicians. Notably, this distribution closely mirrored patterns observed during the pilot phase (i.e., higher nursing adoption than physician adoption), indicating that early adoption preferences persisted during broader implementation.

**Figure 4:**
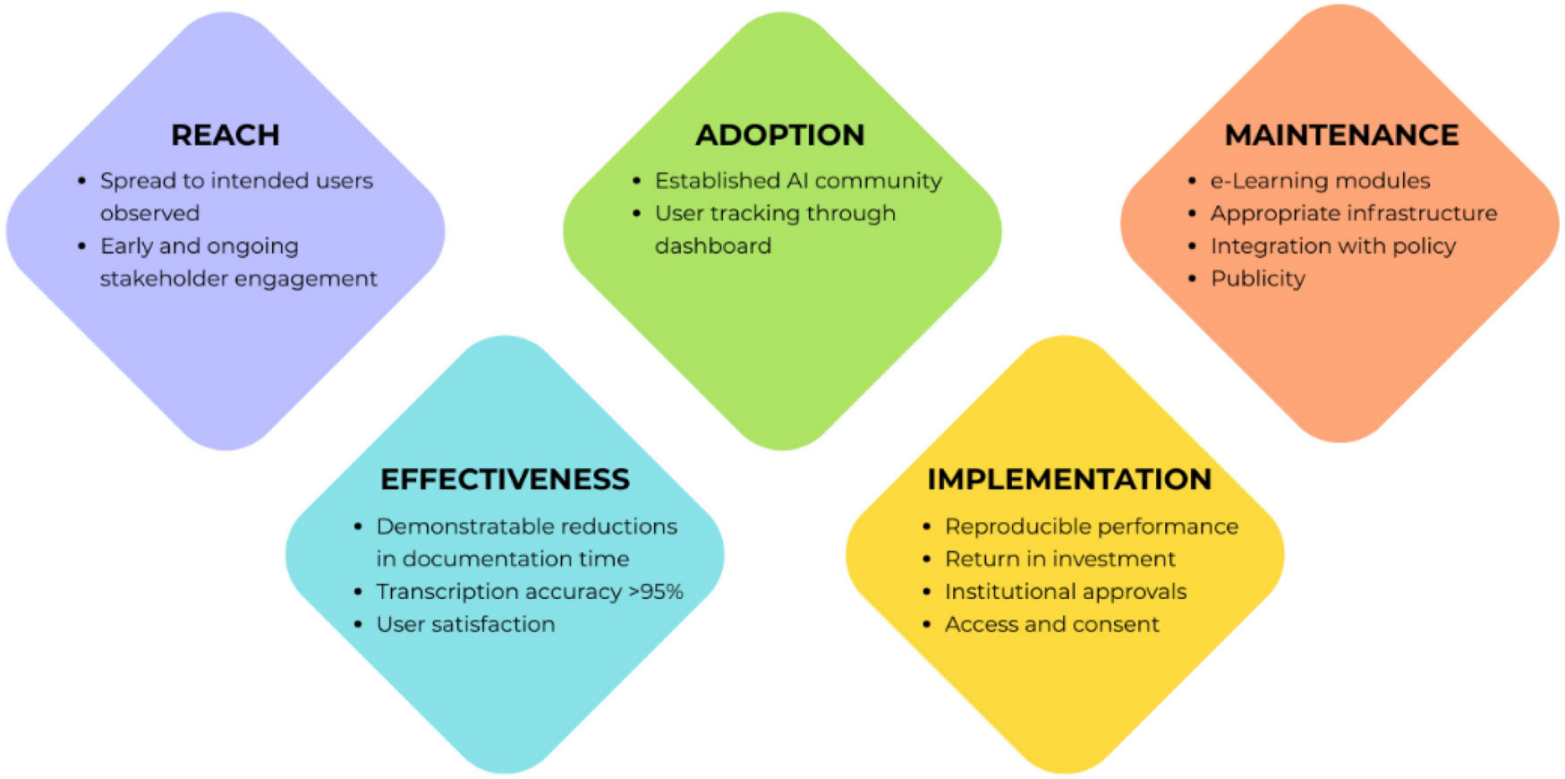
A summary of key inflection points where the transition from pilot to mainstreamed MediVoice use occurred. Data organised using the RE-AIM framework (12).

Scale-up was not uniform. Instead, the transition from pilot to broader adoption unfolded dynamically and was defined by several tipping points (Figure 4) (14). Early and ongoing engagement of both leadership and frontline teams helped establish a strong network of support and ensured MediVoice's relevance to clinical needs. Specific efforts included forming an AI community of practice for users to share experiences and appointing dedicated user champions to promote the tool. During the pilot phase, it was critical to establish value to support broader rollout. Success was defined through several metrics, including measurable reductions in documentation time, high transcription accuracy, and user satisfaction with documentation quality. Other measures included key implementation milestones, such as reproducible system performance across contexts, validation of return on investment, and the establishment of institutional approvals and consent workflows. To further sustain use and embed MediVoice into organisational routines, e-learning modules were introduced, integration with digital transformation roadmaps was undertaken, and continued tracking of spread and sustainment of use was enabled through a user dashboard. Collectively, these strategies created the foundation essential for broader adoption.

## 4 Discussion

The MediVoice journey illustrates the early implementation efforts of an AI scribe within a complex clinical environment. Rapid, iterative testing helped identify several challenges that shaped adoption, including transcription accuracy and speed, and the perceived value across different use cases. These formative experiences reflected a process of collective sense-making, as users and leaders navigated uncertainty through experimentation and feedback. From a state in which cause-and-effect relationships were unclear, to eventual confidence in the system and the establishment of value (15). This involved iterative refinement guided by user feedback to redesign the product while changing workflows. Early disruptions—such as variable performance in multilingual consultations or limited utility in short, templated encounters—became catalysts for organisational learning. These challenges are not technical failures but reflect the capabilities and limitations of ambient AI in practice, reinforcing that uncertainty, adaptation, and clear use-case definitions are integral to its implementation.

Engagement with MediVoice varied across professional groups, reflecting different contexts of use and varying alignment with professional identities. Nurses and allied health professionals adopted the tool more readily than physicians, whose multitasking behaviours reduced the tool's perceived value. This uneven adoption indicates that a one-size-fits-all implementation approach may be suboptimal, as the value proposition lands differently across professions. Such variation underscores the importance of identifying and selecting appropriate use cases that fit within existing workflows and professional identities. It also highlights that certain users may need more support to adapt practice where appropriate. These patterns align with implementation frameworks like the Consolidated Framework for Implementation Research, which highlight how perceived usefulness, attitudes, and social influences can shape adoption (16). Targeted implementation strategies, such as role-specific training and visible leadership endorsement, can help mitigate these barriers to foster trust and skill development (17).

Integration with EMR systems remains a critical next step for routine use, a finding consistent with other digital health implementations (18). However, deployment of ambient AI brings unique challenges. AI scribes (whether integrated or external) are used to generate clinical documentation, upon which care is based. Thus, these systems raise concerns about accuracy, security, and accountability for the content they create. For this reason, the implementation of ambient AI not only requires technical integration but also the establishment of appropriate verification workflows and governance. By adopting a phased and experimental rollout, we allowed time for these processes to evolve. Early engagement with a “good-enough” solution also helped to socialise the approach in clinical practice and accelerate workforce readiness for more advanced applications. Nevertheless, future cost–value evaluations will be essential to ensure that the long-term adoption of ambient AI scribes remains economically and operationally sustainable (19).

Moving forward, sustaining and scaling ambient AI scribes will depend on robust governance and alignment with broader digital health strategies. In Singapore, this means aligning with frameworks issued by the National University Health System's Artificial Intelligence Governance Committee and by the Health Sciences Authority, which emphasise transparency, accountability, and patient safety (20). Complementary international principles further highlight the importance of inclusivity, fairness, and explainability (21). All are important factors to ensure responsible and effective AI use, including AI scribes. In addition to governance, ongoing investment in user education, continued improvements in speech-recognition software, and investment to enhance the availability and quality of audio microphones will be important to ensure more reliable and equitable performance across diverse contexts. Practical measures such as proofreading aids, clear audit trails for text entry, revision and sign-off, and defined liability frameworks will also be essential to build trust and embed AI scribes as safe, reliable components of everyday clinical practice (22, 23).

## 5 Conclusions

Ambient AI has the potential to alleviate the substantial administrative burden faced by healthcare workers today. However, akin to any other AI deployment, successful scale depends on careful attention to usability and integration, organisational readiness, governance structures, and the health systems ability to accommodate uncertainty and flux when deploying new technology.

## Data Availability

The original contributions presented in the study are included in the article/Supplementary Material, further inquiries can be directed to the corresponding authors.
